# Poly[di-μ_2_-chlorido-μ_2_-(1,4-dioxane-κ^2^ *O*:*O*′)-cadmium(II)]

**DOI:** 10.1107/S1600536810048634

**Published:** 2010-11-27

**Authors:** Jian-Qiang Wang, Ren-Jun Du, Wei Wang, Chang-Jun Luan, Cheng Guo

**Affiliations:** a Department of Applied Chemistry, College of Science, Nanjing University of Technology, Nanjing 210009, People’s Republic of China

## Abstract

In the title complex, [CdCl_2_(C_4_H_8_O_2_)]_*n*_, two different  Cd^II^ ions are present, one in a general position and one with site symmetry 2. The Cd^II^ ions are coordinated by two O atoms from two 1,4-dioxane ligands and four chloride anions in a slightly distorted octa­hedral geometry and is connected to neighboring Cd^II^ ions by two bridging chloride anions, generating infinite linear chains along the *a* axis. These chains are further inter­connected by bridging 1,4-dioxane ligands, affording a three-dimensional network.

## Related literature

For background to Cd^II^ complexes, see: Liu *et al.* (2009 ▶); Melnik *et al.* (2009 ▶); Paul *et al.* (2010 ▶); Tatsuya *et al.* (2008 ▶); Xu *et al.* (2009 ▶).
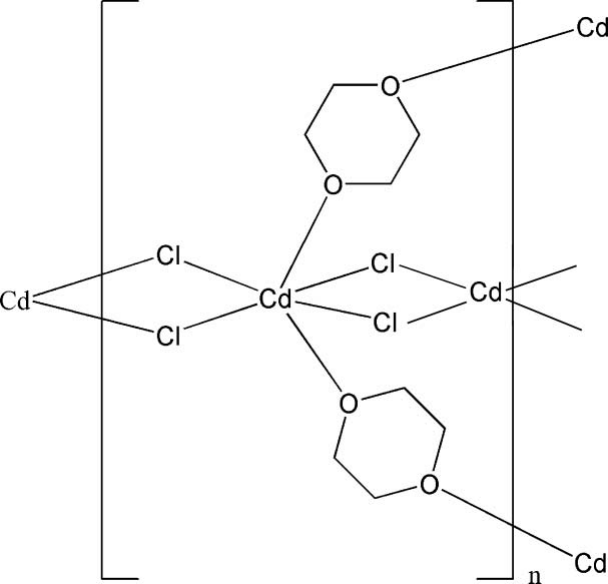


## Experimental

### 

#### Crystal data

[CdCl_2_(C_4_H_8_O_2_)]
*M* *_r_* = 271.40
Monoclinic, 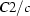

*a* = 15.145 (2) Å
*b* = 13.8871 (18) Å
*c* = 11.5943 (16) Å
β = 102.865 (2)°
*V* = 2377.3 (5) Å^3^
*Z* = 12
Mo *K*α radiation
μ = 3.36 mm^−1^
*T* = 295 K
0.21 × 0.21 × 0.16 mm

#### Data collection

Bruker APEXII CCD diffractometer
Absorption correction: multi-scan (*SADABS*; Sheldrick, 2003 ▶) *T*_min_ = 0.500, *T*_max_ = 0.584
7087 measured reflections
2336 independent reflections
2172 reflections with *I* > 2σ(*I*)
*R*_int_ = 0.018

#### Refinement

*R*[*F* ^2^ > 2σ(*F* ^2^)] = 0.019
*wR*(*F* ^2^) = 0.105
*S* = 1.06
2336 reflections
123 parameters
H-atom parameters constrained
Δρ_max_ = 0.69 e Å^−3^
Δρ_min_ = −0.91 e Å^−3^

### 

Data collection: *APEX2* (Bruker, 2007 ▶); cell refinement: *SAINT* (Bruker, 2007 ▶); data reduction: *SAINT*; program(s) used to solve structure: *SHELXS97* (Sheldrick, 2008 ▶); program(s) used to refine structure: *SHELXL97* (Sheldrick, 2008 ▶); molecular graphics: *SHELXTL* (Sheldrick, 2008 ▶) and *DIAMOND* (Brandenburg, 2005 ▶); software used to prepare material for publication: *SHELXTL*.

## Supplementary Material

Crystal structure: contains datablocks I, global. DOI: 10.1107/S1600536810048634/vm2060sup1.cif

Structure factors: contains datablocks I. DOI: 10.1107/S1600536810048634/vm2060Isup2.hkl

Additional supplementary materials:  crystallographic information; 3D view; checkCIF report

## Figures and Tables

**Fig. 1.**
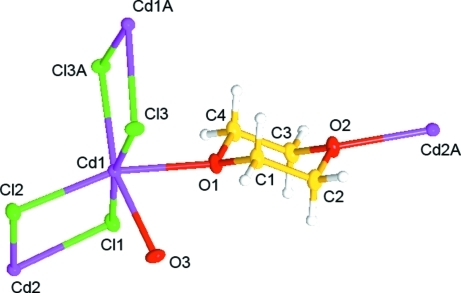
A view of the molecule of (I). Displacement ellipsoids are drawn at the 30% probability level. Symmetry code: (A) -x + 1/2, -y + 1/2, -z + 1.

**Fig. 2.**
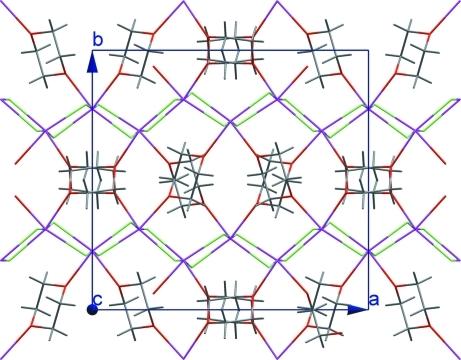
A diagram of the unit cell packing showing the three-dimensional network structure.

## supplementary crystallographic information

### Comment

In the past decade, the design and synthesis of novel cadmium(II) complexes have aroused worldwide interest in the fields of crystal engineering and material chemistry (Melnik *et al.*, 2009). This is due to their intriguing structural features and tailor-made applications as functional materials in chemical catalysis (Paul *et al.*, 2010), gas separation and storage (Liu *et al.*, 2009), luminescence (Tatsuya *et al.*, 2008), and ion-exchange (Xu *et al.*, 2009). During our efforts to investigate the assembly of cadmium(II)-organic coordination frameworks, a new polymer, namely [Cd_3_Cl_6_(dioxane)_3_]_n_, (I), was generated accidentally under normal conditions, and the crystal structure of (I) is described here.

The fundamental building unit of (I) is composed of three Cd^II^ centers, six chlorine anions, and three dioxane ligands. Each Cd^II^ ion adopts a six-coordinated octahedral geometry by coordination to two oxygen donors from two dioxane molecules with Cd—O distances of 2.366 (3) Å and 2.395 (3) Å, and four chlorine atoms with Cd—Cl distances in the range of 2.5628 (10)–2.6252 (11) Å (Fig. 1). Each dioxane ring possesses a chair configuration and bridges two Cd^II^ ions to form an infinite zigzag chain along the [100] direction. Within the zigzag chain, the distance between successive Cd^II^ ions is 7.6093 (8) Å, and the closest Cd—Cd separation between the neighboring strands is 3.7460 (5) Å. Notably, each Cd^II^ ion is also connected with the neighboring Cd^II^ ions by two chlorine anions, thus generating a one-dimensional linear network. These infinite linear chains are further interconnected by bridging dioxane ligands to afford the resulting three-dimensional network (Fig. 2).

### Experimental

A mixture of 1,4-bis(triazol-1-yl-methyl)benzene (48.0 mg, 0.2 mmol) and CdCl_2_.2.5H_2_O (45.7 mg, 0.2 mmol) was dissolved in the dioxane/H_2_O (10 ml, 1:1) solvent media with stirring for *ca* 30 min, and then the resultant colorless solution was filtered. Upon slow evaporation of the filtrate under ambient conditions, block colorless single crystals suitable for X-ray analysis were obtained over a period of two weeks in a yield of 63%. Elemental analysis (%) calcd for C_12_H_24_Cd_3_Cl_6_O_6_: C, 17.70; H, 2.97; Found: C, 17.75; H, 2.97.

### Refinement

All H atoms bound to C atoms were assigned to calculated positions, with C—H = 0.97 Å, and refined using a riding model, with *U*_iso_(H) = 1.2 *U*_eq_(C).

### Crystal data

**Table:**

[CdCl_2_(C_4_H_8_O_2_)]	*F*(000) = 1560
*M**_r_* = 271.40	*D*_x_ = 2.275 Mg m^−^^3^
Monoclinic, *C*2/*c*	Mo *K*α radiation, λ = 0.71073 Å
Hall symbol: -C 2yc	Cell parameters from 5312 reflections
*a* = 15.145 (2) Å	θ = 2.8–30.3°
*b* = 13.8871 (18) Å	µ = 3.36 mm^−^^1^
*c* = 11.5943 (16) Å	*T* = 295 K
β = 102.865 (2)°	Block, colorless
*V* = 2377.3 (5) Å^3^	0.21 × 0.21 × 0.16 mm
*Z* = 12	

### Data collection

**Table:**

Bruker APEXII CCD diffractometer	2336 independent reflections
Radiation source: fine-focus sealed tube	2172 reflections with *I* > 2σ(*I*)
graphite	*R*_int_ = 0.018
φ and ω scans	θ_max_ = 26.0°, θ_min_ = 2.0°
Absorption correction: multi-scan (*SADABS*; Sheldrick, 2003)	*h* = −18→15
*T*_min_ = 0.500, *T*_max_ = 0.584	*k* = −17→17
7087 measured reflections	*l* = −14→14

### Refinement

**Table:**

Refinement on *F*^2^	Primary atom site location: structure-invariant direct methods
Least-squares matrix: full	Secondary atom site location: difference Fourier map
*R*[*F*^2^ > 2σ(*F*^2^)] = 0.019	Hydrogen site location: inferred from neighbouring sites
*wR*(*F*^2^) = 0.105	H-atom parameters constrained
*S* = 1.06	*w* = 1/[σ^2^(*F*_o_^2^) + (0.085*P*)^2^ + 0.3305*P*] where *P* = (*F*_o_^2^ + 2*F*_c_^2^)/3
2336 reflections	(Δ/σ)_max_ = 0.001
123 parameters	Δρ_max_ = 0.69 e Å^−^^3^
0 restraints	Δρ_min_ = −0.91 e Å^−^^3^

### Special details

**Table:**

Geometry. All e.s.d.'s (except the e.s.d. in the dihedral angle between two l.s. planes) are estimated using the full covariance matrix. The cell e.s.d.'s are taken into account individually in the estimation of e.s.d.'s in distances, angles and torsion angles; correlations between e.s.d.'s in cell parameters are only used when they are defined by crystal symmetry. An approximate (isotropic) treatment of cell e.s.d.'s is used for estimating e.s.d.'s involving l.s. planes.
Refinement. Refinement of *F*^2^ against ALL reflections. The weighted *R*-factor *wR* and goodness of fit *S* are based on *F*^2^, conventional *R*-factors *R* are based on *F*, with *F* set to zero for negative *F*^2^. The threshold expression of *F*^2^ > σ(*F*^2^) is used only for calculating *R*-factors(gt) *etc*. and is not relevant to the choice of reflections for refinement. *R*-factors based on *F*^2^ are statistically about twice as large as those based on *F*, and *R*- factors based on ALL data will be even larger.

### Fractional atomic coordinates and isotropic or equivalent isotropic displacement parameters (Å^2^)

**Table:**

	*x*	*y*	*z*	*U*_iso_*/*U*_eq_	
Cd1	0.330474 (14)	0.189847 (18)	0.421324 (19)	0.02267 (15)	
Cd2	0.5000	0.27411 (3)	0.2500	0.02297 (16)	
C1	0.3234 (2)	0.4003 (3)	0.0979 (3)	0.0326 (8)	
H1A	0.2736	0.3744	0.1282	0.039*	
H1B	0.3363	0.3559	0.0391	0.039*	
C2	0.3835 (3)	0.4763 (3)	0.2794 (3)	0.0301 (8)	
H2A	0.4365	0.4832	0.3435	0.036*	
H2B	0.3344	0.4514	0.3121	0.036*	
C3	0.4236 (2)	−0.0363 (3)	0.4210 (3)	0.0285 (8)	
H3A	0.3812	−0.0333	0.3449	0.034*	
H3B	0.4074	−0.0909	0.4642	0.034*	
C4	0.4829 (2)	0.0496 (3)	0.5972 (3)	0.0299 (8)	
H4A	0.4685	−0.0025	0.6457	0.036*	
H4B	0.4795	0.1096	0.6387	0.036*	
C5	0.2025 (3)	−0.0029 (3)	0.4581 (3)	0.0311 (8)	
H5A	0.1532	0.0221	0.4904	0.037*	
H5B	0.2554	−0.0097	0.5225	0.037*	
C6	0.1427 (3)	0.0732 (3)	0.2772 (3)	0.0339 (9)	
H6A	0.1552	0.1178	0.2186	0.041*	
H6B	0.0930	0.0989	0.3081	0.041*	
Cl1	0.35989 (6)	0.16305 (8)	0.20972 (8)	0.0297 (2)	
Cl2	0.47678 (7)	0.29108 (8)	0.46654 (8)	0.0297 (2)	
Cl3	0.30007 (7)	0.18952 (6)	0.63572 (8)	0.0278 (2)	
O1	0.40258 (16)	0.41052 (19)	0.1933 (2)	0.0291 (6)	
O2	0.41779 (16)	0.05092 (19)	0.4859 (2)	0.0307 (6)	
O3	0.22192 (17)	0.06271 (19)	0.3719 (2)	0.0314 (6)	

### Atomic displacement parameters (Å^2^)

**Table:**

	*U*^11^	*U*^22^	*U*^33^	*U*^12^	*U*^13^	*U*^23^
Cd1	0.0198 (2)	0.0212 (2)	0.0288 (2)	0.00012 (9)	0.00928 (14)	−0.00097 (9)
Cd2	0.0207 (2)	0.0212 (3)	0.0288 (2)	0.000	0.00941 (16)	0.000
C1	0.0313 (19)	0.032 (2)	0.0322 (18)	0.0103 (16)	0.0017 (15)	−0.0036 (16)
C2	0.0341 (19)	0.029 (2)	0.0252 (16)	0.0101 (16)	0.0032 (15)	−0.0018 (15)
C3	0.0250 (18)	0.0258 (18)	0.0333 (18)	0.0023 (15)	0.0035 (15)	−0.0029 (15)
C4	0.0287 (18)	0.033 (2)	0.0264 (16)	0.0106 (16)	0.0029 (14)	−0.0025 (15)
C5	0.0317 (19)	0.036 (2)	0.0258 (16)	−0.0106 (17)	0.0077 (15)	0.0006 (15)
C6	0.036 (2)	0.028 (2)	0.0342 (19)	−0.0066 (17)	−0.0002 (16)	0.0032 (16)
Cl1	0.0298 (5)	0.0317 (5)	0.0299 (4)	−0.0078 (4)	0.0115 (4)	−0.0060 (4)
Cl2	0.0284 (5)	0.0333 (5)	0.0285 (5)	−0.0085 (4)	0.0083 (4)	−0.0019 (4)
Cl3	0.0282 (5)	0.0283 (5)	0.0291 (5)	0.0062 (3)	0.0113 (4)	0.0015 (3)
O1	0.0286 (13)	0.0291 (15)	0.0285 (12)	0.0113 (11)	0.0043 (10)	−0.0019 (11)
O2	0.0263 (13)	0.0311 (15)	0.0313 (13)	0.0114 (11)	−0.0012 (10)	−0.0072 (11)
O3	0.0309 (14)	0.0279 (14)	0.0319 (13)	−0.0110 (12)	0.0000 (11)	0.0023 (11)

### Geometric parameters (Å, °)

**Table:**

Cd1—O2	2.364 (2)	C2—H2B	0.9700
Cd1—O3	2.393 (2)	C3—O2	1.439 (4)
Cd1—Cl3^i^	2.5634 (10)	C3—C4^iv^	1.489 (5)
Cd1—Cl2	2.5775 (10)	C3—H3A	0.9700
Cd1—Cl1	2.6158 (10)	C3—H3B	0.9700
Cd1—Cl3	2.6269 (10)	C4—O2	1.438 (4)
Cd2—O1	2.401 (2)	C4—C3^iv^	1.489 (5)
Cd2—O1^ii^	2.401 (2)	C4—H4A	0.9700
Cd2—Cl1	2.5804 (10)	C4—H4B	0.9700
Cd2—Cl1^ii^	2.5804 (10)	C5—O3	1.431 (4)
Cd2—Cl2^ii^	2.6222 (10)	C5—C1^v^	1.506 (5)
Cd2—Cl2	2.6222 (10)	C5—H5A	0.9700
C1—O1	1.446 (4)	C5—H5B	0.9700
C1—C5^iii^	1.506 (5)	C6—O3	1.442 (4)
C1—H1A	0.9700	C6—C2^v^	1.511 (5)
C1—H1B	0.9700	C6—H6A	0.9700
C2—O1	1.429 (4)	C6—H6B	0.9700
C2—C6^iii^	1.511 (5)	Cl3—Cd1^i^	2.5634 (10)
C2—H2A	0.9700		
			
O2—Cd1—O3	77.28 (9)	O1—C2—H2B	109.7
O2—Cd1—Cl3^i^	163.70 (7)	C6^iii^—C2—H2B	109.7
O3—Cd1—Cl3^i^	88.35 (7)	H2A—C2—H2B	108.2
O2—Cd1—Cl2	89.19 (7)	O2—C3—C4^iv^	110.4 (3)
O3—Cd1—Cl2	164.70 (7)	O2—C3—H3A	109.6
Cl3^i^—Cd1—Cl2	105.95 (4)	C4^iv^—C3—H3A	109.6
O2—Cd1—Cl1	88.92 (6)	O2—C3—H3B	109.6
O3—Cd1—Cl1	85.52 (6)	C4^iv^—C3—H3B	109.6
Cl3^i^—Cd1—Cl1	97.68 (3)	H3A—C3—H3B	108.1
Cl2—Cd1—Cl1	87.14 (3)	O2—C4—C3^iv^	111.0 (3)
O2—Cd1—Cl3	84.39 (6)	O2—C4—H4A	109.4
O3—Cd1—Cl3	88.25 (6)	C3^iv^—C4—H4A	109.4
Cl3^i^—Cd1—Cl3	87.58 (3)	O2—C4—H4B	109.4
Cl2—Cd1—Cl3	97.60 (3)	C3^iv^—C4—H4B	109.4
Cl1—Cd1—Cl3	171.72 (3)	H4A—C4—H4B	108.0
O1—Cd2—O1^ii^	75.83 (12)	O3—C5—C1^v^	110.0 (3)
O1—Cd2—Cl1	89.52 (7)	O3—C5—H5A	109.7
O1^ii^—Cd2—Cl1	162.08 (7)	C1^v^—C5—H5A	109.7
O1—Cd2—Cl1^ii^	162.08 (7)	O3—C5—H5B	109.7
O1^ii^—Cd2—Cl1^ii^	89.52 (7)	C1^v^—C5—H5B	109.7
Cl1—Cd2—Cl1^ii^	106.59 (5)	H5A—C5—H5B	108.2
O1—Cd2—Cl2^ii^	82.66 (6)	O3—C6—C2^v^	109.5 (3)
O1^ii^—Cd2—Cl2^ii^	89.20 (6)	O3—C6—H6A	109.8
Cl1—Cd2—Cl2^ii^	99.24 (3)	C2^v^—C6—H6A	109.8
Cl1^ii^—Cd2—Cl2^ii^	86.95 (3)	O3—C6—H6B	109.8
O1—Cd2—Cl2	89.20 (6)	C2^v^—C6—H6B	109.8
O1^ii^—Cd2—Cl2	82.66 (6)	H6A—C6—H6B	108.2
Cl1—Cd2—Cl2	86.95 (3)	Cd2—Cl1—Cd1	92.87 (3)
Cl1^ii^—Cd2—Cl2	99.24 (3)	Cd1—Cl2—Cd2	92.79 (3)
Cl2^ii^—Cd2—Cl2	169.69 (5)	Cd1^i^—Cl3—Cd1	92.42 (3)
O1—C1—C5^iii^	109.5 (3)	C2—O1—C1	109.6 (3)
O1—C1—H1A	109.8	C2—O1—Cd2	121.4 (2)
C5^iii^—C1—H1A	109.8	C1—O1—Cd2	119.1 (2)
O1—C1—H1B	109.8	C4—O2—C3	110.4 (3)
C5^iii^—C1—H1B	109.8	C4—O2—Cd1	121.2 (2)
H1A—C1—H1B	108.2	C3—O2—Cd1	128.02 (19)
O1—C2—C6^iii^	109.9 (3)	C5—O3—C6	109.3 (3)
O1—C2—H2A	109.7	C5—O3—Cd1	122.7 (2)
C6^iii^—C2—H2A	109.7	C6—O3—Cd1	121.3 (2)
			
O1—Cd2—Cl1—Cd1	−85.54 (6)	Cl1—Cd2—O1—C1	−36.6 (2)
O1^ii^—Cd2—Cl1—Cd1	−50.8 (2)	Cl1^ii^—Cd2—O1—C1	117.8 (3)
Cl1^ii^—Cd2—Cl1—Cd1	102.42 (3)	Cl2^ii^—Cd2—O1—C1	62.8 (2)
Cl2^ii^—Cd2—Cl1—Cd1	−168.02 (3)	Cl2—Cd2—O1—C1	−123.6 (2)
Cl2—Cd2—Cl1—Cd1	3.68 (3)	C3^iv^—C4—O2—C3	57.2 (4)
O2—Cd1—Cl1—Cd2	−92.98 (7)	C3^iv^—C4—O2—Cd1	−116.2 (3)
O3—Cd1—Cl1—Cd2	−170.31 (7)	C4^iv^—C3—O2—C4	−56.9 (4)
Cl3^i^—Cd1—Cl1—Cd2	101.97 (4)	C4^iv^—C3—O2—Cd1	116.0 (3)
Cl2—Cd1—Cl1—Cd2	−3.74 (3)	O3—Cd1—O2—C4	−137.6 (3)
O2—Cd1—Cl2—Cd2	92.64 (6)	Cl3^i^—Cd1—O2—C4	−108.9 (3)
O3—Cd1—Cl2—Cd2	65.1 (2)	Cl2—Cd1—O2—C4	49.6 (2)
Cl3^i^—Cd1—Cl2—Cd2	−93.49 (4)	Cl1—Cd1—O2—C4	136.8 (2)
Cl1—Cd1—Cl2—Cd2	3.68 (3)	Cl3—Cd1—O2—C4	−48.1 (2)
Cl3—Cd1—Cl2—Cd2	176.87 (3)	O3—Cd1—O2—C3	50.2 (3)
O1—Cd2—Cl2—Cd1	85.83 (7)	Cl3^i^—Cd1—O2—C3	78.9 (3)
O1^ii^—Cd2—Cl2—Cd1	161.64 (7)	Cl2—Cd1—O2—C3	−122.6 (3)
Cl1—Cd2—Cl2—Cd1	−3.73 (3)	Cl1—Cd1—O2—C3	−35.4 (3)
Cl1^ii^—Cd2—Cl2—Cd1	−110.05 (4)	Cl3—Cd1—O2—C3	139.7 (3)
O2—Cd1—Cl3—Cd1^i^	−165.80 (7)	C1^v^—C5—O3—C6	60.0 (4)
O3—Cd1—Cl3—Cd1^i^	−88.42 (7)	C1^v^—C5—O3—Cd1	−148.7 (2)
Cl3^i^—Cd1—Cl3—Cd1^i^	0.0	C2^v^—C6—O3—C5	−59.7 (4)
Cl2—Cd1—Cl3—Cd1^i^	105.77 (4)	C2^v^—C6—O3—Cd1	148.5 (2)
C6^iii^—C2—O1—C1	−59.4 (4)	O2—Cd1—O3—C5	58.0 (3)
C6^iii^—C2—O1—Cd2	155.4 (2)	Cl3^i^—Cd1—O3—C5	−114.2 (3)
C5^iii^—C1—O1—C2	59.2 (4)	Cl2—Cd1—O3—C5	86.3 (3)
C5^iii^—C1—O1—Cd2	−154.7 (2)	Cl1—Cd1—O3—C5	147.9 (3)
O1^ii^—Cd2—O1—C2	−64.2 (2)	Cl3—Cd1—O3—C5	−26.6 (3)
Cl1—Cd2—O1—C2	105.4 (3)	O2—Cd1—O3—C6	−154.0 (3)
Cl1^ii^—Cd2—O1—C2	−100.2 (3)	Cl3^i^—Cd1—O3—C6	33.8 (3)
Cl2^ii^—Cd2—O1—C2	−155.2 (3)	Cl2—Cd1—O3—C6	−125.7 (3)
Cl2—Cd2—O1—C2	18.4 (3)	Cl1—Cd1—O3—C6	−64.1 (2)
O1^ii^—Cd2—O1—C1	153.8 (3)	Cl3—Cd1—O3—C6	121.4 (3)

Symmetry codes: (i) −*x*+1/2, −*y*+1/2, −*z*+1; (ii) −*x*+1, *y*, −*z*+1/2; (iii) −*x*+1/2, *y*+1/2, −*z*+1/2; (iv) −*x*+1, −*y*, −*z*+1; (v) −*x*+1/2, *y*−1/2, −*z*+1/2.
